# ECCFP: A consecutive full pass‐based bioinformatic analysis for eccDNA identification from long‐read sequencing data

**DOI:** 10.1002/imo2.70080

**Published:** 2026-02-03

**Authors:** Wang Li, Biyuan Miao, Jun Zhang, Qingsong Zeng, Tangxuan Zhang, Zetong Wu, Yusheng Song, Mengting Li, Lilu Guo, Juan Luo, Jingxiang Xu, Tianliang Liu, Shifu Chen, Jia Gu, Shaogui Wan

**Affiliations:** ^1^ Institute of Genomics and Precision Medicine, School of Medical Technology, Gannan Medical University Ganzhou China; ^2^ School of Medical and Information Engineering, Gannan Medical University Ganzhou China; ^3^ School of Basic Medicine, Gannan Medical University Ganzhou China; ^4^ Department of Interventional Radiology Ganzhou Hospital‐Nanfang Hospital, Southern Medical University (Ganzhou People's Hospital) Ganzhou China; ^5^ Institute of Neurology, Jiangxi Academy of Clinical Medical Sciences, The First Affiliated Hospital, Jiangxi Medical College, Nanchang University Nanchang China; ^6^ Department of Computer Science Xidian University Xi'an China; ^7^ LifeX Institute, School of Medical Technology, Gannan Medical University Ganzhou China; ^8^ Department of Data Science City University of Macau Macau China

**Keywords:** consecutive full pass, ECCFP, extrachromosomal circular DNA, ghost sequence, long‐read sequencing

## Abstract

Emerging studies focused on extrachromosomal circular DNA (eccDNA) due to its various functions and complex mechanisms. The main technique for eccDNA detection is using high‐throughput sequencing followed by bioinformatics analysis, but with shortcomings such as low accuracy and efficiency. To address these limitations, we developed ECCFP, a bioinformatics pipeline that detects eccDNAs from long‐read sequencing data with rolling circle amplification. ECCFP retains all consecutive full passes from individual reads, including those from ghost sequences, to obtain the candidate eccDNAs. Subsequently, ECCFP employs the Boyer–Moore majority voting algorithm to consolidate the multiple candidate eccDNAs to generate an accurate unique eccDNA and circular consensus sequence. By using multiple simulated datasets and real sequencing datasets with spike‐in eccDNAs or publicly available, we conducted comprehensive evaluations of ECCFP and compared it with three currently published pipelines that have demonstrated superior performance. The results indicate that ECCFP outperforms other pipelines in sensitivity, accuracy, and runtime efficiency. Furthermore, outward PCR amplification followed by Sanger sequencing validation confirmed that ECCFP detects more accurate eccDNA junction position than other pipelines. Conclusively, ECCFP is an efficient and accurate bioinformatic pipeline for eccDNA detection from long‐read sequencing data, which might provide a robust tool for eccDNA characterization in various biological samples.

## Highlights

- ECCFP is an innovative tool that utilizes all consecutive full passes from individual reads and the Boyer–Moore voting algorithm to improve the eccDNA detection.
- ECCFP exhibits advantages in overall performance, sensitivity, and accuracy with high runtime efficiency, compared to other pipelines.
- ECCFP is an open‐source project available at https://github.com/WSG-Lab/ECCFP, featuring easy installation and comprehensive usage tutorials.

## Graphical abstract

A new bioinformatics pipeline called ECCFP has been developed to improve the detection of extrachromosomal circular DNA (eccDNA) from long‐read sequencing data. ECCFP uses all consecutive full passes from individual reads for candidate eccDNA identification and consolidates candidate eccDNAs to generate accurate unique eccDNA. To thoroughly evaluate the performance of ECCFP, 6 simulated datasets, 2 real sequencing datasets with spiked‐in artificial synthesized eccDNAs and 29 publicly available real sequencing datasets, were applied. The results demonstrate that ECCFP markedly reduces the false positive rate, significantly increases the number of unique eccDNAs detection, achieves higher accuracy in identifying junction positions and circular consensus sequences, and also shortens the runtime, when compared to other three eccDNA analysis pipelines. Overall, ECCFP provides a more efficient and accurate bioinformatic analysis for eccDNA detection from long‐read sequencing data, which might push the research forward on eccDNA as a biomarker in clinical settings.

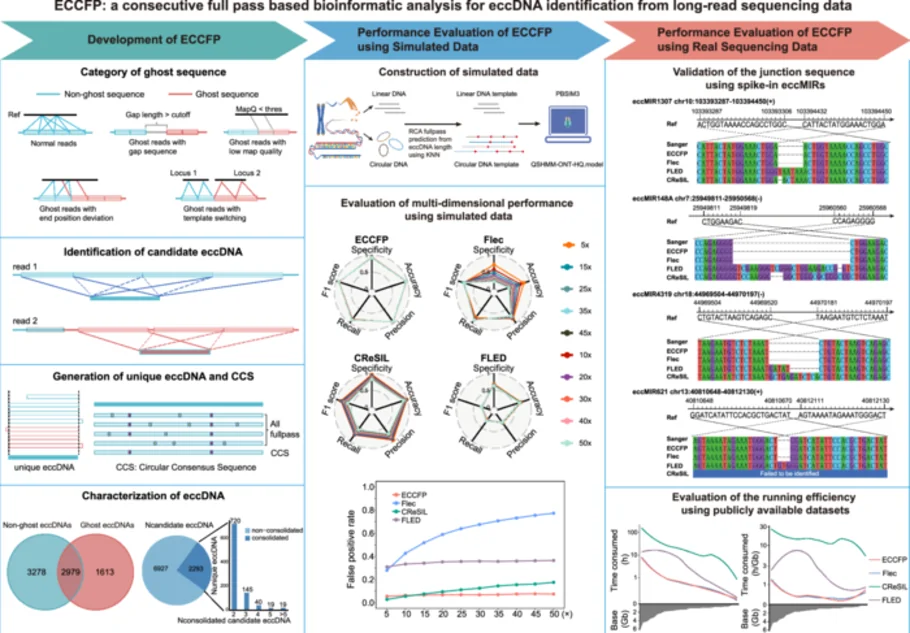

## 1 INTRODUCTION

Extrachromosomal circular DNA (eccDNA) is a type of circular DNA that exists independently of chromosomes and is widely distributed in eukaryotes, including humans, animals, plants, and fungi [1]. Owing to the relative stability of its circular topology, eccDNA undergoes chromosome‐extrinsic amplification and recombination, driving copy‐number variation and gene rearrangements, or functioning as mobile regulatory elements that involve gene expression regulation [2]. Consequently, eccDNA is increasingly recognized to contribute to many pathological or physiological processes, especially in tumorigenesis, tumor evolution of heterogeneity and therapeutic resistance [3, 4]. Recently, eccDNA has garnered broad attention and shows a wide variety of functions in precision medicine [5], crop breeding [6], aging assessment [7], immunomodulation [8], and prenatal screening [9]. Notably, it was documented that eccDNA in body fluids could be a promising biomarker for liquid biopsy and longitudinal disease monitoring [10].

Commonly, eccDNA is categorized by length or origin into microRNA carrying circular DNA (microDNA), small polydisperse circular DNA (spcDNA), telomeric circles (t‐circles), and extrachromosomal circular DNA (ecDNA) [11]. Among these, ecDNA specifically refers to circular DNA molecules larger than 100 kilobases (kb), and it has been detected in nearly half of cancer types and plays a critical role in tumor evolution [12]. Given the size variation, two strategies are typically used for eccDNA detection. For large‐size ecDNA, visualization approaches, such as FISH and microscopy, and unenriched high‐throughput sequencing methods, such as whole genome sequencing (WGS), ATAC‐seq, and CRISPR‐CATCH, are used for circular DNA characterization [13]. For small‐to‐intermediate eccDNA identification, RCA is commonly used for circular DNA enrichment, followed by high‐throughput sequencing and bioinformatics analysis. Accumulating evidence indicates that small eccDNAs are markedly enriched in repetitive sequences, which may serve as indicators of replication stress and genome instability across aging and disease contexts [3]. Currently, eccDNA detection usually relies on short‐read Next‐Generation Sequencing (NGS) platforms, followed by bioinformatic analysis, such as Circle‐Map [14]. Since most eccDNA likely originates repetitive sequences, short‐read NGS‐based sequencing technology has difficulty accurately identifying chimeric eccDNAs and eccDNAs derived from tandem repeat regions.

Recently, long‐read Third‐Generation Sequencing (TGS) has been increasingly employed for the detection of eccDNA [15]. Although the long‐read TGS‐based technology showed significant advantages for complex repetitive sequence characterization of eccDNA, which overcomes the limitation of short‐read NGS‐based technology, the bioinformatic analysis for TGS data still has many challenges for eccDNA characterization [13, 16, 17]. For instance, ecc_finder [18] has low sensitivity, CiderSeq2 [19] is not suitable for the identification of complex eukaryotes, and cyrcular‐calling [20] imposes more stringent high‐depth data. The eccDNA_RCA_nanopore (full‐length eccDNA caller, Flec) [8, 21] identifies eccDNA based on tandem repeats within each single read; however, its single‐read approach might lead to an increase in false‐positive rate. CReSIL [22] filters false positives by selecting the largest genomic region, which significantly overestimates eccDNA boundaries and might result in inaccurate eccDNA position. FLED [23] employs stringent thresholds using a 70% read‐mapping rate, which might result in significant data loss during processing. By the way, among all the current TGS‐based bioinformatic pipelines, Flec, CReSIL, and FLED can output complete eccDNA sequences, and their overall performance is relatively superior in the current studies [13]. Collectively, although the TGS‐based eccDNA bioinformatics pipelines were emerging recently, the limitations mentioned above highlight the need for further optimization of current eccDNA pipelines in terms of accuracy and efficiency.

To address the limitations of eccDNA identification from TGS‐based data, here we developed a novel pipeline named EccDNA Caller based on Consecutive Full Pass (ECCFP) and comprehensively evaluated the performance comparing with current eccDNA bioinformatics pipelines, including Flec, CReSIL, and FLED. Our results suggested that ECCFP detected a greater number of eccDNA molecules, achieved higher junction position accuracy, and exhibited superior runtime efficiency.

## 2 RESULTS

### EccDNA identification workflow

Prior to initiating the ECCFP workflow, raw data were obtained from an RCA‐enriched eccDNA library with sequencing on Oxford Nanopore Technology (ONT) PromethION 2 platform (Figure 1A) and were followed by preprocess to generate an alignment file (mapping.paf) that serves as the input data (Figure 1B). The ECCFP pipeline consists of four major steps to identify accurate eccDNA and output the results, including circular consensus sequences (CCS) and variants (Figure 1C).

**FIGURE 1:**
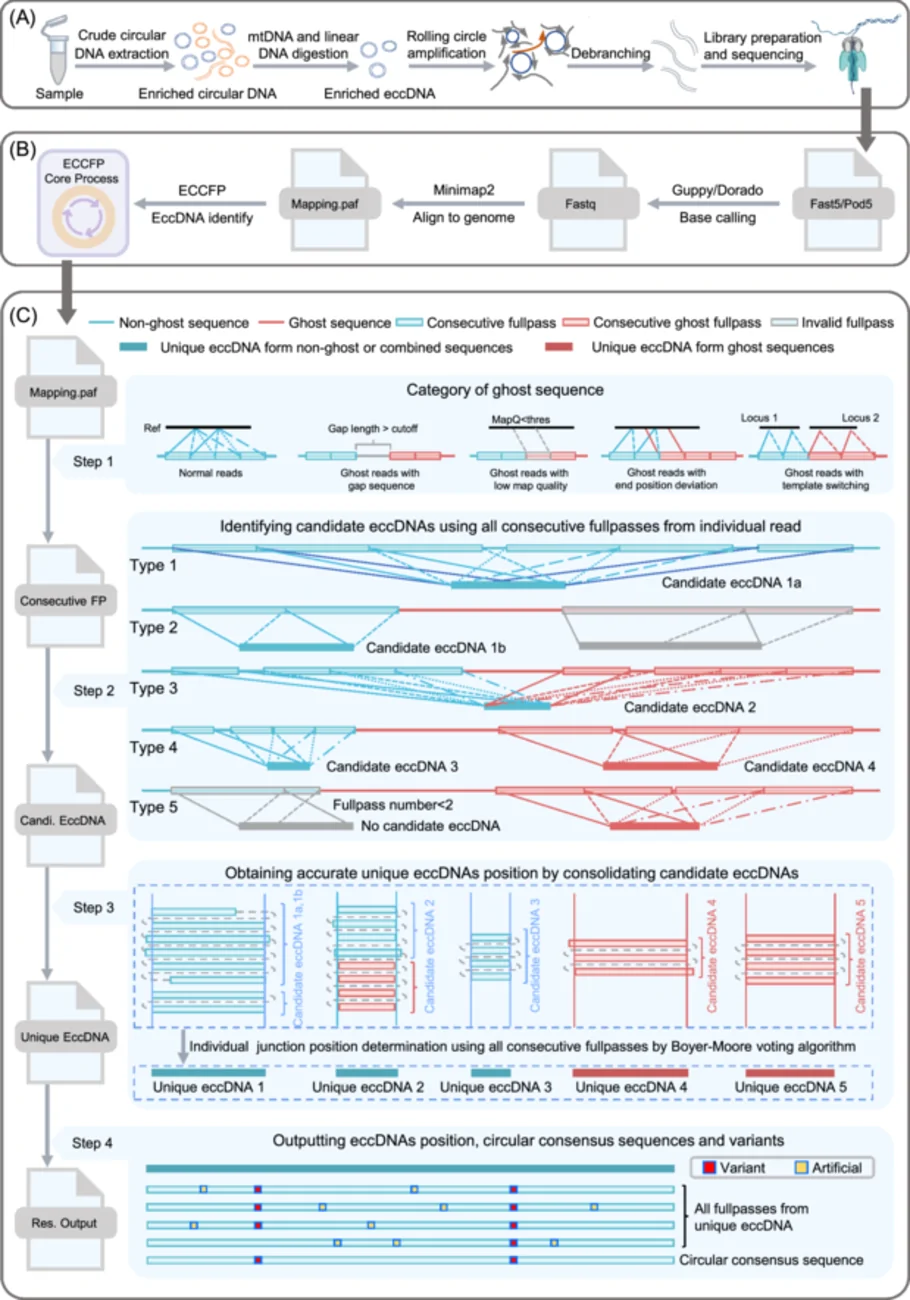
Workflow of eccDNA identification by EccDNA Caller based on Consecutive Full Pass (ECCFP). (A) Experiment process of circular DNA enrichment, library preparation and nanopore sequencing. (B) Data preparation for ECCFP analysis. Base calling through Guppy or Dorado, followed by alignment to the reference genome with Minimap2. (C) The core process of ECCFP. **Step 1**: This step demonstrates the category of ghost sequence used for ECCFP analysis. Ideally, normal reads should align continuously and with high quality to the reference genome, but the real reads are often more complex due to the RCA process and sequencing error. Excepting for normal reads, other reads that contain consecutive full pass will be classified as ghost reads in any of the following situations: (1) With unaligned gaps in the reads that exceed the threshold length; (2) There are subreads with alignment quality below the mapping quality of threshold; (3) The subreads align to the near region of the genome as the previous full passes but exhibit a significant deviation at their end positions; (4) The subreads align to different regions of the genome compared to the previous full passes. **Step 2:** Identifying candidate eccDNAs using all consecutive full passes from individual read. Based on the candidate eccDNA identification, the reads can be categorized into the following five types: Type 1: Candidate eccDNA is identified from reads that do not contain ghost sequences; Type 2: Candidate eccDNA is identified only from non‐ghost sequences, although the read contains ghost sequences with invalid full pass; Type 3: The identical candidate eccDNAs are identified from both ghost and non‐ghost sequences in an individual read; Type 4: The different candidate eccDNAs are identified from ghost and non‐ghost sequences in an individual read, respectively; Type 5: The candidate eccDNA is identified only from ghost sequences. **Step 3:** Obtaining an accurate unique eccDNA position by consolidating candidate eccDNAs. All identified candidate eccDNAs are further processed, and the final unique eccDNA is obtained using the Boyer–Moore voting algorithm. **Step 4:** Results output. Outputting the unique eccDNA position, aligning all full passes sequences to obtain the accurate consensus sequence and variants calling.

In step 1, ECCFP uses the alignment file mapping.paf to trim tandem repeat units from individual reads. Overlapping regions between repeat units are removed to prevent redundant use of overlapping sequences, while gaps between units are preserved. To avoid potential artifacts from non‐eccDNA sequences, the gaps are not allowed to be filled using reference genome sequences. In an ideal situation, a read should align continuously and with high quality to the reference genome. However, due to situations such as the RCA process and random errors in real sequencing, the real sequencing data might be much more complex. During alignment, when reads contain any of the following conditions: (1) having an unaligned gap sequence exceeding the threshold length (default: 20 bp); (2) exhibiting low mapping quality (default: MapQ < 30); (3) showing a deviation of the mapped terminal position from the reference genome that exceeds the preset threshold length (default: 20 bp); (4) or displaying evidence of template switching, the subsequent reads that include full passes will be classified as ghost sequences.

In step 2, candidate eccDNAs are identified. Consecutive full passes (≥2 full‑passes) are defined as tandemly repeated sequences within a single read that align to the same chromosome region and serve as candidate eccDNA identification. Based on the eccDNAs identified from individual reads, each read was classified into one of the following types: Type 1: Reads containing only non‑ghost consecutive full‑passes; Type 2: Reads that contain ghost sequences, from which eccDNAs are identified only from the non‑ghost regions; Type 3: Reads containing both non‑ghost and ghost sequences, with both regions identified as the same eccDNA; Type 4: Reads containing both non‑ghost and ghost sequences, with each region identified as different eccDNAs; Type 5: Reads in which eccDNAs are identified exclusively from ghost sequences. Ultimately, all consecutive full passes from the same chromosomal location are consolidated into a single candidate eccDNA. Notably, template switching during RCA may result in multiple candidate eccDNAs from a single read. All candidate eccDNAs and their corresponding supporting full pass information are maintained in the intermediate file unit.csv.

In step 3, unique eccDNAs are obtained. Candidate eccDNAs derived from different reads but mapped to the same chromosomal region were consolidated by the Boyer–Moore voting algorithm if their start and end positions deviated by no more than a preset threshold (default: 20 bp) and shared a consistent orientation. The final junction position is determined by selecting genomic coordinates supported by the maximum number of full passes supporting. ECCFP improves the precise positioning of eccDNA through this voting strategy, significantly reducing false positives. The information on consolidation is maintained in the intermediate file candidate_consolidated.csv. To reflect RCA‐derived molecular authenticity, the pipeline retains eccDNAs with divergent orientations at identical positions while maintaining fidelity to the original full passes observed in sequencing reads. These orientation‐specific results are provided in the output file final_eccDNA.csv for user‐defined filtration.

In step 4, construct a CCS and variant calling. ECCFP generates CCS from all consecutive full passes used for identification. Considering the random errors inherent in nanopore sequencing, a nucleotide is designated as a mutational event only when both the minimum sequencing depth threshold (default: 4×) and the mutant base frequency threshold (default: 75%) occur simultaneously. Otherwise, the pipeline reverts to the reference base at that position. Ultimately, ECCFP generates the following three output files: final_eccDNA.csv, consensus_sequence.fasta, and variant.csv.

### Increasing the number of eccDNAs detection by ECCFP

During the RCA process, amplification‐induced errors increase the complexity of reads, leading to the formation of five major read types (Figure 1C, step2). Current pipelines typically retain only the longest alignment segment starting from the beginning of the read, while discarding the other parts as ghost sequences [22]. By retaining all consecutive full passes in a full‐length read, which includes ghost sequences, ECCFP achieves maximal data utilization while effectively suppressing the generation of erroneous sequences. In addition, the selection of reads with a full pass count of two or more further enhances data accuracy and alleviates the computational burden.

In the sequencing data of the HepG2 cell line, 30,184 reads with a full pass ≥2 were included in the analysis. If ghost sequences were discarded, as in current pipelines, only 60.6% of the available data would be fully utilized (Figure 2A). Our results showed that ghost sequences also exhibit high base quality comparable to non‐ghost sequences (Figure 2B). Further comparison of the five read types revealed that type 3 reads were more susceptible to sequencing quality than the others (Figure 2C). Moreover, comparisons of length distribution and chromosomal distribution revealed that there were no significant differences between ghost and non‐ghost sequences. (Figure 2D,E). Ghost sequences were predominantly distributed among types 3, 4, and 5 (Figure 2F). EccDNA molecules identified from different read types showed partial overlaps (Figure 2G). Additionally, ECCFP successfully identified 4592 eccDNA molecules from ghost sequences, including 1613 newly identified eccDNA molecules (Figure 2H). The newly identified eccDNAs exclusively originated from read types 4 and 5, comprising 851 and 675 eccDNA molecules (Figure 2I). Furthermore, we performed Sanger sequencing to validate the eccDNA molecules that were identified exclusively in the ghost sequences. Remarkably, the junction sequence validation confirmed that all 10 selected eccDNAs were present in the sequencing library, and their sequencing results were fully consistent with the identified sequences (Figure 2J). These findings indicate that, under stringent quality control conditions, retaining ghost sequences significantly increased the number of detected eccDNA molecules.

**FIGURE 2:**
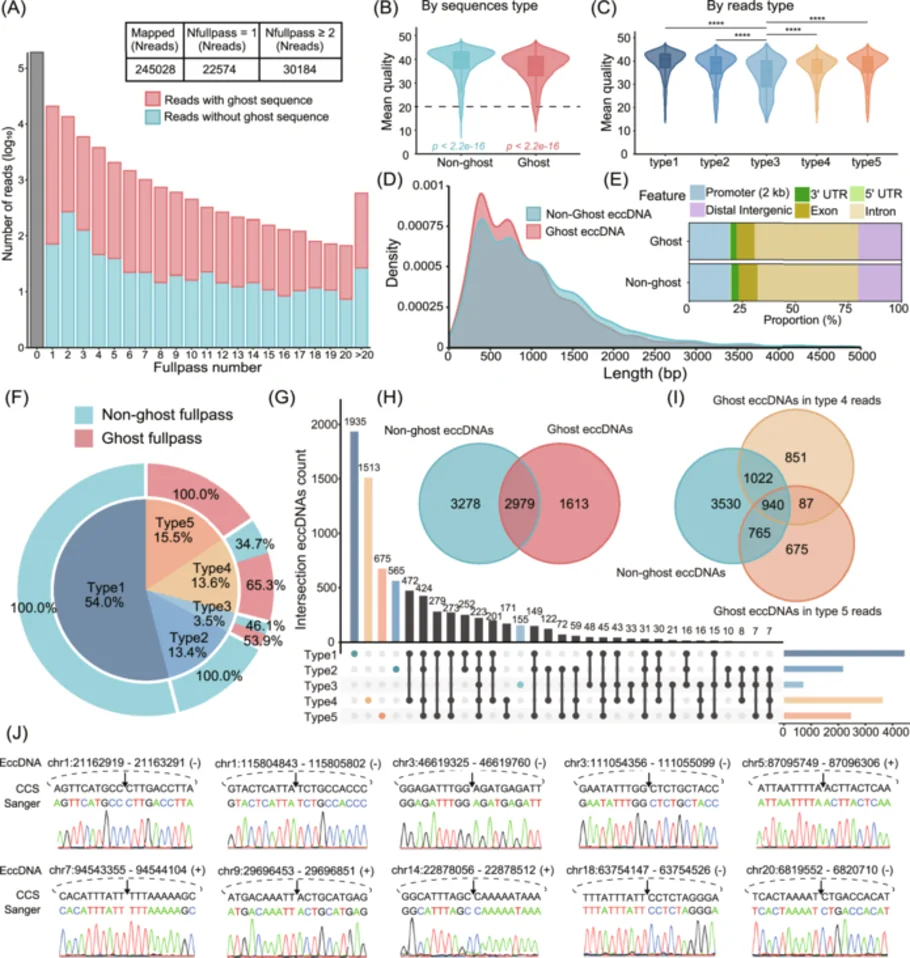
The number of eccDNA detection by ECCFP. (A) A total of 245,028 mapped reads from the sequencing data of the HepG2 cell line were aligned to the reference genome (GRCh38.p14), with 30,183 reads (Nfullpass ≥ 2) being used for the eccDNA identification. (B) Distribution of base quality scores for non‐ghost sequences and ghost sequences. One‐sided Wilcoxon signed‐rank tests were conducted, with *H*_0_: M ≤ 20 versus *H*_1_: M > 20, where *M* denotes the median quality score and is indicated as a dash line. (C) Distribution of mean quality scores for five types of reads for eccDNA identification. The Mann–Whitney *U* test was used to compute *p*, which were adjusted for multiple comparisons using the Holm–Bonferroni method. (D) Size distribution of eccDNA identified from non‐ghost and ghost sequences. (E) Gene annotation of eccDNA identified from non‐ghost and ghost sequences. (F) Proportion of full pass sources among the five types of reads identifying eccDNA. (G) Number of unique eccDNA identified by the five types of reads. The horizontal bar chart represents the number of eccDNA identified by each read type, while the vertical bar chart shows the intersection of eccDNA among the different read types. (H) Venn diagram showing the intersection of total eccDNA derived from non‐ghost sequences and ghost sequences. (I) Venn diagram showing the increased newly identified eccDNAs from ghost sequence only in type 4 and type 5 reads. (J) The junction sequence validation of 10 selected eccDNAs exclusively derived from ghost sequences was performed with outward PCR followed by Sanger sequencing. The arrow indicates the junction position of eccDNA consensus sequence.

### Improving the accuracy of eccDNA position characterization by ECCFP

To obtain more accurate position and reduce false positives of eccDNA detection, ECCFP consolidated the candidate eccDNAs using the Boyer–Moore majority voting algorithm to obtain accurate unique eccDNA positions and CCS (Figure 1C, step 3). Taking the eccDNA^chr12:109862500‐109864540^ as an example, ECCFP successfully identified 7 candidate eccDNAs with various supporting read coverage (Figure 3A), which were subsequently consolidated into a unique eccDNA by the majority voting process (Figure 3B). Sanger sequencing further validated the consistency of the junction sequence coordinates, suggesting the accuracy of the detection by ECCFP (Figure 3B). Consequently, candidate eccDNAs can be categorized into two groups: consolidated and non‐consolidated. In the sequencing data of the HepG2 cell line, a total of 9220 candidate eccDNAs were identified. After consolidating, the number of eccDNAs was reduced to 7870 unique eccDNAs, with 2293 candidate eccDNAs being consolidated into 943 unique eccDNAs, which means that ECCFP eliminates 1350 potential false‐positive results (Figure 3C).

**FIGURE 3:**
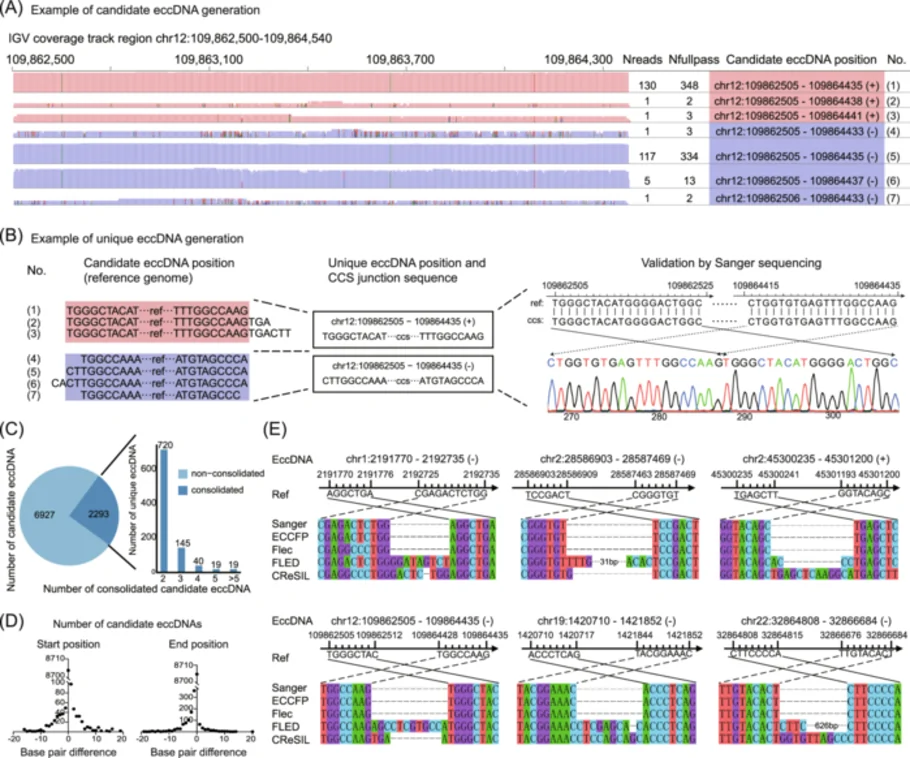
The position of eccDNA characterization by EccDNA Caller based on Consecutive Full Pass (ECCFP). The accurate, unique eccDNAs junction position was identified by the Boyer–Moore majority voting algorithm through all consecutive full passes and consolidation of candidate eccDNAs. (A) Example of candidate eccDNA generation. Using the genomic region of chr12:109,862,500–109,864,540 as an example, seven candidate eccDNAs with different ends and strand orientations (positive strand indicated with light coral and negative strand indicated with light blue) can be obtained based on the ECCFP identification results of individual reads. Only one position has a significant accumulation of reads for the two different strands forming a circle, while the results with different ends are considered false positives. (B) Example of unique eccDNA generation. Based on the seven candidate eccDNAs obtained in panel (A), we performed a majority voting process on their ends, weighted by Nfullpass, ultimately resulting in a unique eccDNA at the identical position from each strand. Further validation by Sanger sequencing confirmed the accurate position of this unique eccDNA. (C) The pie chart displays the number of candidate eccDNAs categorized as consolidated and unconsolidated, while the bar chart shows the quantity of unique eccDNAs with varying numbers of consolidated candidate eccDNAs. (D) Number of candidate eccDNAs corresponding to base pair differences between the total unique (position 0) and candidate eccDNAs (varying base pair difference). (E) The comparison of junction sequence identified by ECCFP and other eccDNA analysis pipelines. Six unique eccDNA were selected for Sanger sequencing validation, and the results of the junction sequence were presented using MEGA.

The base pair difference between the start and end positions of the unique eccDNAs and their corresponding candidate eccDNAs indicates that ECCFP is capable of obtaining the positions of eccDNA with high accuracy (Figure 3D). To assess the accuracy of ECCFP, we compared the junction sequence detection of six selected eccDNAs by the current eccDNA analysis pipelines. Using Sanger sequencing of outward PCR products as a reference (Figure S1A), we found that ECCFP and Flec identified consistent end positions, while FLED and CReSIL showed inaccurate results (Figure 3E). Although Flec can detect the correct position, its output is only at the single‐read level, and the errors introduced by RCA amplification and sequencing may cause the same eccDNAs to exhibit slight alignment deviations across multiple reads, leading to redundant results [17]. These results indicate that the eccDNA position identified by ECCFP corresponds more closely to the actual genomic region in the sequencing data, which presents the real eccDNA on the genome (Table S1).

### Evaluating the performance of ECCFP by spiking in artificially synthesized circular DNA

To further assess the accuracy of eccDNA detection by ECCFP, we artificially synthesized four circular DNAs and added them to the crude extract of the HepG2 cell line, respectively as eccMIR621 (1483 bp, approximately 33.33 fg of 21,500 copy number), eccMIR4319 (694 bp, approximately 11.11 fg of 14,600 copy number), eccMIR148A (758 bp, approximately 3.7 fg of 4450 copy number) and eccMIR1307(1164 bp, approximately 1.23 fg of 964 copy number). Concurrently, we removed linear DNA and mitochondrial DNA from the unaltered HepG2 cell line, followed by RCA and sequencing (Figure 4A). Both groups of sample data showed high quality (Figure 4B). By employing four pipelines to analyze the sequencing data, it was observed that ECCFP can identify more eccDNA molecules compared to the other three pipelines (Figure 4C). The correlation analysis between the eccDNA supporting reads number or full pass number and the input copy number of the eccMIR was performed, and the results revealed a strong correlation for most pipelines (Figure 4D–G). Notably, the correlation between the reads number given by ECCFP and the input quantity was the highest (Figure 4E). It is worth mentioning that CReSIL could not identify eccMIR621, which was spike‐in with the highest amount, in any of the three samples (Figure 4H). To further validate the four spike‐in eccDNAs, we performed verification through outward PCR amplification and Sanger sequencing (Figure S1B). Consensus sequences that closely matched the Sanger results were selected from each analysis pipeline and compared using MEGA software. Consistent with the previously detected results, ECCFP output eccDNA CCS sequences were completely identical to the Sanger sequencing results, while CReSIL and FLED results still displayed deviations in start and end positions and sequence content (Figure 4H). Similarly, the identification results from Flec exhibited a significant amount of redundancy. These findings further indicate that ECCFP not only identifies more eccDNA molecules but also significantly reduces false positives while maintaining high accuracy and reliability.

**FIGURE 4:**
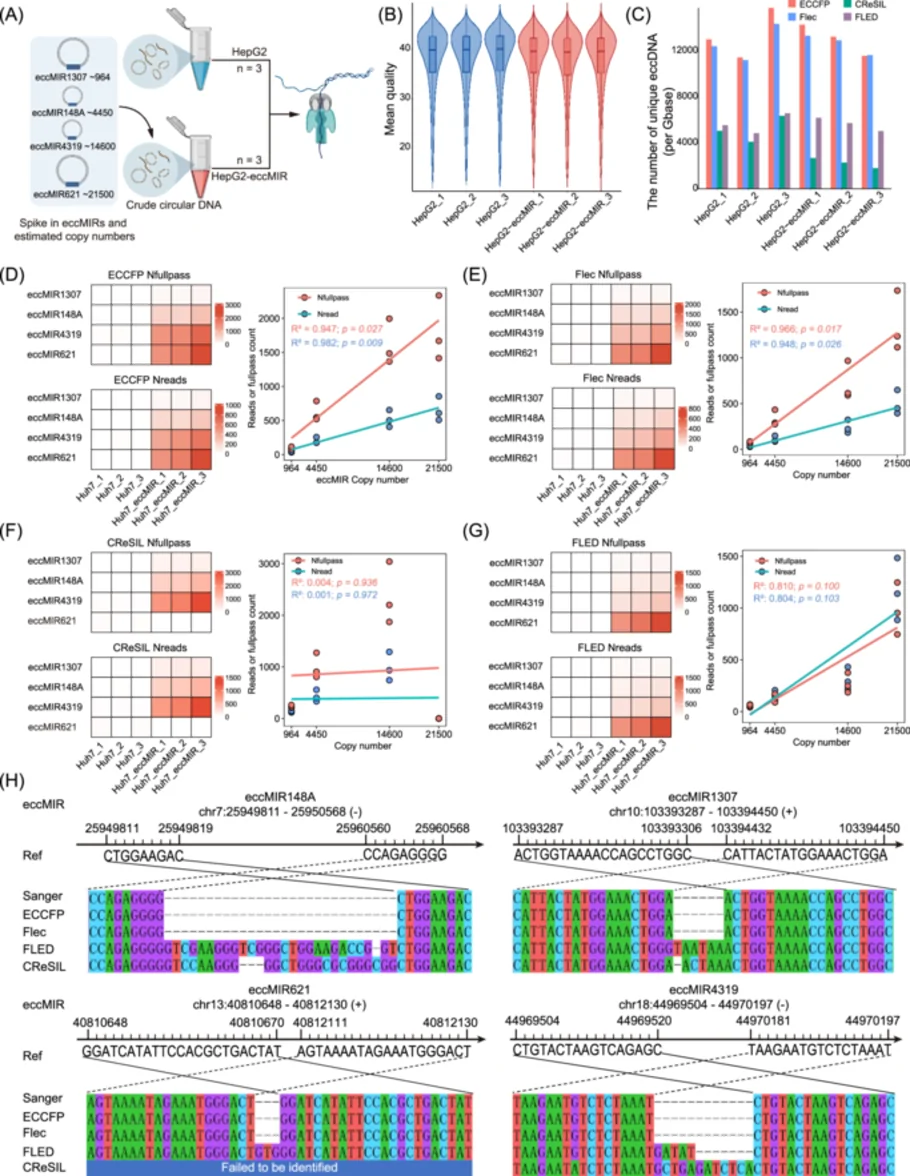
Evaluation of eccDNA detection by ECCFP using spiking in artificially synthesized circular DNA. (A) Four synthesized eccMIRs with various copies were spiked into the crude extract of circular DNA sample from HepG2 cell line. Sample preparation, library construction, and sequencing were performed in triplicate for both spike‐in eccMIRs and control HepG2 samples. (B) Mean base quality distribution across two group samples. (C) Number of unique eccDNA identified by four eccDNA analysis pipelines, normalized to per Gbase. Heatmaps and correlation analyses of 4 spik‐in eccMIRs identified by ECCFP (D), Flec (E), CReSIL (F), and FLED (G) pipelines are presented. The CReSIL pipeline misidentified eccMIR621, which has the highest copy number. (D–G) Evaluate the correlation between spike‐in eccMIR copy number and the corresponding eccDNA detection amounts using Pearson's correlation coefficient. (H) Comparison of junction sequence of four spike‐in eccMIRs identified by different pipelines and validated with Sanger sequencing.

### Evaluating the performance of ECCFP using simulated data

We generated a simulated dataset (Figure 5A) along with the publicly available simulated datasets [17, 22] to comprehensively assess the performance of ECCFP and compare it with other pipelines, including Flec, CReSIL, and FLED. When generating the simulated data, we considered the characteristics of the data produced by RCA. We evaluated the proportion of read types based on ghost sequences in samples from public databases, and the simulated data generated from this assessment accurately reflects the real data situation encountered during the real sequencing process of libraries constructed based on RCA. By assessing the read types in the downloaded public simulated datasets, we confirmed our hypothesis (Figure S2A). Although these simulated datasets mimic the real sequencing process, they still cannot fully represent the reality of ghost sequences present in the real data.

**FIGURE 5:**
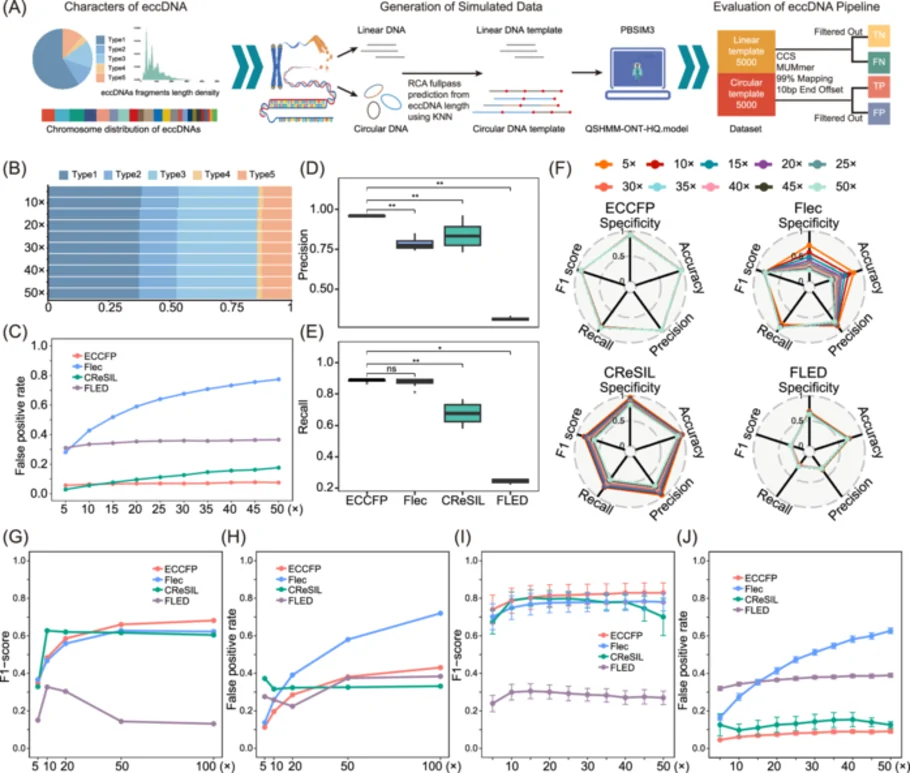
The performance evaluation of ECCFP using simulated data. (A) Construction process for simulated data, based on the proportions of five types of reads, eccDNA length distribution, and chromosomal distribution. TP (True Positive): An eccDNA identified by the pipeline that aligns to a simulated circular sequence with >99% identity and <10 bp positional deviation (strict criterion). FP (False Positive): An eccDNA identified by the pipeline that does not meet the above criteria. FN (False Negative): A simulated circular sequence not detected by the pipeline. TN (True Negative): A simulated linear sequence correctly identified as non‐eccDNA. Detailed information about each simulated dataset is provided in Table S4. (B) Proportions of the five types of reads at different simulated data depths. Inter‐method false positive rates (C), precision (D), and recall (E) analyses were conducted using the simulated data constructed in this study. For (D, E), where data are paired, *p* were calculated with the Wilcoxon signed‐rank test and subsequently adjusted for multiple testing using the Holm‐Bonferroni correction. (F) Multi‐dimensional performance analysis among four eccDNA analysis pipelines evaluated using simulated data constructed in this study. F1 score (G) and false positive rate (H) comparisons among four eccDNA analysis pipelines evaluated using simulated data constructed by Wanchai et al. 2022 [22]. F1 score (I) and false positive rate (J) comparisons among four eccDNA analysis pipelines evaluated using simulated data constructed by Gao et al. 2024 [17].

It is worth noting that previous studies typically employed relatively relaxed conditions of 90% sequence homology and a terminal deviation of 20 bp. The results showed that relaxed conditions may lead to an overestimation of the pipelines' performance (Figures S3A–E and S4A). To evaluate a more accurate comparison of the performance differences among various pipelines, we adopted stricter standards of 99% sequence homology and a terminal deviation of 10 bp (Figures 5C–J, S2B,C, and S4A,B) for the evaluation. When comparing the four pipelines using various depth simulated data with the similar read type ratios (Figure 5B), ECCFP demonstrated the most stable and superior performance. At different depths, the false positive rate of ECCFP remains consistently low, and no significant increase is observed as depth increases (Figure 5C). In addition, ECCFP demonstrates excellent performance in the evaluation of precision and recall (Figure 5D,E). The F1‐score of ECCFP remains stable above 0.9 across various depths of simulated data. Moreover, ECCFP achieved better comprehensive scores when analyzing low‐depth sequencing data (Figure 5F). Furthermore, when evaluated using other simulated eccDNA datasets that do not take ghost sequences into account, ECCFP also demonstrated excellent performance, with the false positive rate generally remaining at a low level (Figure 5G–J). These findings emphasize the broader applicability and stability of ECCFP (Table S2).

### Evaluating the performance of ECCFP using public RCA‐based nanopore sequencing data

We downloaded 29 RCA‐based nanopore sequencing datasets from various organism sample types, including 10 human tumor cell lines [22, 23], 3 mouse tumor cell lines [22], 2 mouse tumor tissues [22], 12 Arabidopsis thaliana seeds [24], and 2 tomatoes seeds [15], to thoroughly assess the eccDNA detection performance of ECCFP, and compare it with other eccDNA analysis pipelines (Figure 6A, Table S3). After performing size and quality statistics for all datasets, we found that data of various sizes also exhibited high quality in ghost sequences (Figure 6B,C). Furthermore, we compared the number of eccDNAs detected and the runtime across different pipelines using the same dataset. The results clearly demonstrate that ECCFP requires a shorter runtime while achieving superior efficiency (Figure 6D,E). In most datasets, ECCFP can identify more unique eccDNAs (Figure 6F), approximately 2%–11% of eccDNAs that are not identified by the other pipelines (Figure 6G). When setting the criteria to 90% sequence identity for reproducibility analysis of eccDNA analysis pipelines, ECCFP shows the greatest overlap with other pipelines, which covers 60% to 93% of the unique eccDNA from the other pipelines (Figure 6H, Table S4). These results indicate that ECCFP can detect a greater number of unique eccDNAs in a shorter runtime, demonstrating consistent and excellent performance across different species with reference genomes.

**FIGURE 6:**
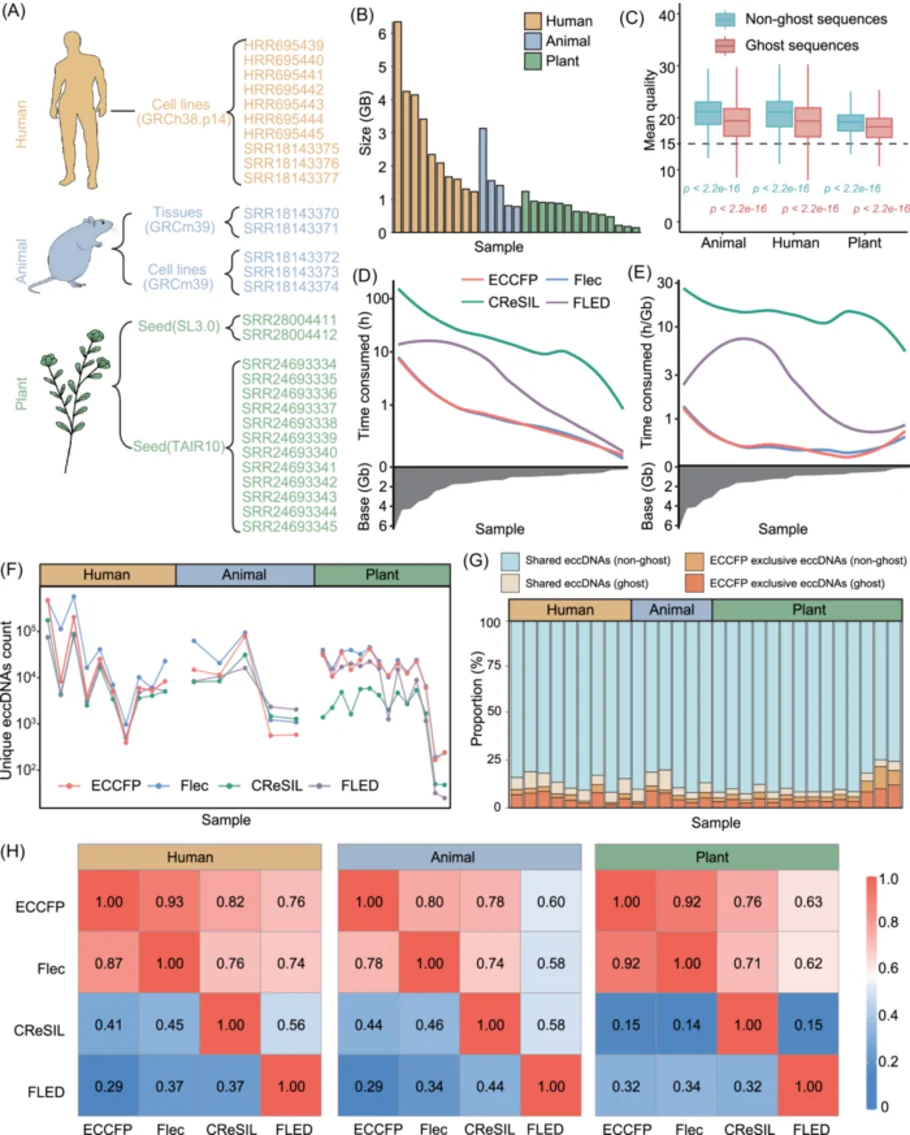
The performance evaluation of ECCFP using publicly available datasets. (A) Sample source of public datasets. (B) The sizes of the sequencing data of the public datasets by organism types. (C) Mean base quality distribution of ghost and non‐ghost sequences across different types of organisms. One‐sided Wilcoxon signed‐rank tests were performed, with *H*_0_: M ≤ 15 versus *H*_1_: M > 15, where M denotes the median quality score and indicates as dash line. Assessment of runtime (D) and normalized runtime (E) for eccDNA identification using four eccDNA analysis pipelines among the public datasets, with the *x*‐axis arranged by data size from high to low. (F) Number of unique eccDNAs identified by four eccDNA analysis pipelines among the public datasets. (G) Assessment of unique eccDNA detection between ECCFP and other eccDNA analysis pipelines. Shared eccDNAs are those identified by all pipelines, while ECCFP exclusive eccDNAs are those identified exclusively by ECCFP. (H) Heatmap of reproducibility of eccDNA identification by four eccDNA analysis pipelines. 90% homology threshold of unique eccDNA CCS was applied for the heatmap analysis.

## 3 DISCUSSION

Based on comprehensive comparisons of the currently bioinformatics pipelines, including Flec, CReSIL, and FLED, ECCFP, as a novel eccDNA identification pipeline for RCA‐based long‐read sequencing data, exhibits significant advantages over the current pipelines due to its novelty strategy and algorithms (Table S5). This new pipeline not only accurately identifies the locations of eccDNA and collects CCS information but also efficiently utilizes data while significantly reducing runtime.

In this study, we innovatively employed ghost sequences in the identification process, demonstrating that these sequences, which are filtered with strict requirements, can actually be effectively utilized to identify additional eccDNAs. Under ideal conditions, the products of RCA are characterized by tandem repeat units observed in sequencing reads [25, 26]. However, amplification errors introduced during the RCA process increase the complexity of the reads. Owing to the potential template switching by phi29 DNA polymerase during RCA [27], the generation of highly branched RCA products that may interrupt sequencing and produce erroneous long reads [22], as well as the random errors that can occur during long‐read sequencing, these artifacts are often recognized as ghost sequences and subsequently may be excluded by other pipelines. We found that the quality of ghost sequences available for identification does not noticeably decline after rigorous filtering (Figure 2B). Through outward PCR and Sanger validation, the authenticity of the identified eccDNAs from ghost sequences was confirmed, significantly enhancing the effective data utilization rate with the same amount of sample template and thereby significantly improving the efficiency of eccDNA detection.

Moreover, by implementing a voting algorithm strategy, ECCFP can achieve a more precise identification of eccDNA junction positions. This approach demonstrates superior performance in comparison and validation, outperforming current eccDNA analysis pipelines. For example, while Flec demonstrates a strong capability in detecting diverse eccDNA molecules, it produces a high proportion of redundant results [17], leading to numerous false positives. This redundancy compromises detection reproducibility and introduces biases in downstream analyses. CReSIL and FLED also exhibit limitations in the accuracy of start and end positions and in the stability of CCS generation.

We conducted comparative validation of ECCFP performance by spiking in four artificial synthetic eccMIRs with known sequences as a “gold standard” dataset. Our results indicate that ECCFP can detect junction positions with greater accuracy and displays a closer correlation with the actual spike‐in quantities. However, the other three pipelines performed less effectively in this respect. We found that CReSIL integrates eligible fragment regions within the analysis process, ultimately outputting a maximum size of chromosomal coordinates. Therefore, in regions with higher sequencing depth, the merged area may exceed the preset threshold (default of 50 bp), resulting in an incomplete full pass across the entire merged region. This situation could lead to undetected cases, even for the highest copy number spike‐in eccMIR621 (Figure 4F). Our results suggest that ECCFP can serve as an indicator for estimating eccDNA molecular quantity. However, due to the specificity of RCA, the quantities of detected full passes and reads can provide only a conservative estimate.

To more rigorously evaluate the performance of eccDNA analysis pipelines, we generated simulated datasets to reflect the real sequencing data based on RCA and downloaded the independent simulated datasets from online sources. ECCFP exhibits robust and stable performance across three datasets under both relaxed and stringent standards, with higher F1 scores at different sequencing depths. Under stringent standards, Flec shows a significant decline in precision and specificity as sequencing depth increases, along with an increase in the false positive rate. Currently, performance evaluation is primarily conducted through two methods: coordinate homology and sequence homology. FLED performs relatively well when assessed using coordinate homology (Figure S4A,B). However, its overall performance in sequence homology evaluations, even with relaxed standards, is unsatisfactory (Figure S3). We found that the number of CCS outputted by FLED is lower than the number of detected eccDNAs, which may contribute to its poor performance. Overall, CReSIL demonstrates strong and stable performance across various datasets and standards; however, its F1 score exhibits a downward trend with increasing sequencing depth (Figures 5I, S3E), which is consistent with the result of spik‐in eccMIR621, suggesting that higher sequencing depth may lead to a higher incidence of undetected cases.

When evaluated on multiple real sequencing datasets from various organism samples, ECCFP consistently showed overall advantages. It achieved the highest correlation with eccDNA molecules identified by other pipelines and detected more eccDNA molecules while also minimizing redundancy. These results demonstrate the robustness of ECCFP across different species and types based on long‐read sequencing data generated by RCA. However, among the 29 samples analyzed, we observed that three samples (HRR695445, SRR18143373, and SRR18143374) yielded fewer identified eccDNA molecules compared with the results from the other three pipelines. Further examination revealed that these samples contained a higher proportion of reads with fewer than two full passes (Table S3), suggesting these samples do not have enough reads containing consecutive full passes for eccDNA identification by ECCFP.

Although employing a full pass ≥2 threshold for eccDNA identification enhances data reliability, computational efficiency, and memory performance, it might also be a limitation of ECCFP in some low‐quality sequencing datasets with a low proportion of consecutive full passes. Moreover, during eccDNA enrichment, the RCA step exhibits a bias toward amplifying eccDNA molecules shorter than 10 kb [28]. Consequently, RCA‐based methods are better suited for the detection of small eccDNA species, such as microDNA and spcDNA, while being less effective for identifying large ecDNA molecules. This is another limitation of ECCFP, which is unable to detect large sizes of eccDNA.

Conclusively, ECCFP, as a novel bioinformatic pipeline, is specifically designed to identify small sizes of eccDNA from long‐read sequencing data with RCA amplification. ECCFP combines the advantages of current eccDNA analysis pipelines, and it is optimized for the characteristics of reads amplified by RCA. It demonstrates significant advantages in sensitivity, accuracy, and operational efficiency (Table S5). In recent years, as research on eccDNA has progressed, small eccDNAs have been shown to play important roles in nature. Furthermore, accurate detection of eccDNA will provide robust support for research across disciplines, driving progress in related areas. ECCFP provides a novel tool to advance both basic research and clinical translation. The application of ECCFP for accurate eccDNA detection might facilitate the transition of eccDNA research from mechanistic exploration to clinical biomarker applications.

## 4 CONCLUSION

In this study, we present a new bioinformatics pipeline, named ECCFP, to improve the detection of extrachromosomal circular DNA from long‐read sequencing data. ECCFP uses all consecutive full passes from individual reads for candidate eccDNA identification and consolidates candidate eccDNAs to generate accurate unique eccDNA. Overall, ECCFP provides a more efficient and accurate bioinformatic analysis for eccDNA detection from long‐read sequencing data, which might advance research on eccDNA as a biomarker in clinical settings.

## 5 METHODS

### Cell cultivation and crude circular DNA isolation

The human hepatoma cell line HepG2 was obtained from Procell Life Science & Technology (Cat No: CL‐0103). HepG2 cells were cultured in minimum essential medium supplemented with 10% fetal bovine serum and 1% penicillin‐streptomycin. The HepG2 cells were harvested during the exponential growth phase. Approximately 2 × 10^6^ cells were washed twice with cold phosphate buffer saline at pH 7.4. Crude circular DNA extraction was then performed using the E.Z.N.A. Endo‐free Plasmid Mini Kit (omega) according to the manufacturer's instructions. The concentration of the extracted DNA was determined using a Qubit 4.0 (Thermo Fisher Scientific).

### Artificial synthesis of circular DNA

Artificial circular DNA was synthesized according to the method of ligase‐assisted mini‐circle accumulation (LAMA) [29]. Briefly, using the target eccDNA as a template, two semi‐complementary linear DNA fragments (linear A and linear B) are produced via PCR. Then, Taq DNA Ligase (NEB) is used to randomly ligate semi‐complementary linear A and linear B fragments into circular DNA molecules. Finally, the residual linear DNA in the LAMA reaction product is digested with Exonuclease V (NEB) at 37°C overnight. The resulting circular DNA is then purified from the hydrolysis products using VAHTS DNA Clean Beads (Vazyme). Four circular DNA molecules (eccMIR621, eccMIR4319, eccMIR148A and eccMIR1307) were synthesized and spiked into the crude circular DNA extract of HepG2 cells at gradient concentrations of 33 fg, 11 fg, 3.7 fg, and 1.3 fg, respectively.

### EccDNA enrichment and RCA amplification

To linearize mitochondrial DNA, 10 µL of extracted HepG2 DNA was digested with the genomic rare‐cutting endonuclease Quick Digest MssI (Thermo Fisher Scientific) at 37°C for 6 h, then heat‐inactivated at 65°C for 10 min. Linear DNA was subsequently removed with Exonuclease V (NEB) at 37°C for 6 h, followed by inactivation at 70°C for 10 min. After digesting linear and mitochondrial DNA, qPCR was used to confirm the presence of linear chromosomal and mitochondrial DNA in the product. The digested crude circular DNA sample was extracted with DNA extraction solution (ACMEC) in a Phase Lock Gel tube (TIANGEN), followed by ethanol precipitation to enrich eccDNA.

Subsequently, following the manufacturer's recommended protocol, we performed RCA with random primers (Thermo Fisher Scientific) using Phi29 DNA polymerase (Thermo Fisher Scientific). The reaction mixture was incubated at 30°C for 6 h, followed by incubation at 65°C for 5 min. Subsequently, the RCA product was subjected to additional treatment using T7 endonuclease (NEB) at 37°C for 15 min to remove branching structures. The DNA concentration was measured using the Qubit 4.0 fluorometer.

### Library preparation and sequencing

Debranched DNA was employed as input, following the manufacturer's instructions, for the sequencing library construction using the Native Barcoding Kit 24 V14 (ONT, SQK‐NBD114.24). The library was sequenced on PromethION instrument using a flow cell (R10.4.1, FLO‐PRO114M) following the manufacturer's instructions.

### Bioinformatic analysis

The raw data were basecalled using Dorado (v7.9.8). Subsequently, adaptor and barcode sequences were removed using Porechop to generate clean reads in FASTQ format. Quality control of the FASTQ files was performed using Nanoplot [30], followed by alignment of the reads to the reference genome GRCh38.p14 using Minimap2 [31] (‐cx map‐ont ‐‐secondary = no ‐t 16) to generate an alignment file (mapping.paf) that serves as the input. Then, ECCFP‐based identification of candidate eccDNAs was performed on reads containing at least two full passes, which were derived from RCA‐based long‐read sequencing data. The Boyer–Moore voting algorithm was used to obtain unique eccDNA.

EccDNA identification and result filtering were conducted using ECCFP, Flec [8, 21], CReSIL [22], and FLED [23] according to their respective guidelines, and overlaps between identification results from different pipelines were performed using bedtools intersect [32]. Specifically, eccDNA results from Flec required a full pass ≥2, results from CReSIL required classification as *ctc*, and results from FLED required classification as *Full*. The number, length distribution, and chromosomal distribution of eccDNAs were visualized using R. CCS generated by different pipelines were compared using MEGA software.

### Verification of eccDNA junction sequence by outward PCR and Sanger sequencing

The outward PCR primers were designed containing the junction sequence of the eccDNA for PCR amplification (Table S6). Qubit 4.0 was used for quantification, and fragment distribution was detected by the S2 cartridge (Qsep100 advance). The PCR products were subsequently subjected to Sanger sequencing validation (Sangon Biotech).

### Simulated data generation and performance evaluation

The simulated eccDNA sequences were designed to mimic the size and chromosomal distribution of eccDNA sequencing data from public databases, as well as to reference the proportions of different types of reads in real RCA‐based sequencing data. A Python‐based program was utilized to generate 5000 circular sequences (as positives) and 5000 linear sequences (as negatives) from the hg38 reference genome. The program incorporated a K‐nearest neighbors (KNN) algorithm to simulate the number of RCA cycles, ensuring alignment with real sequencing data distributions. We regenerated the simulation data according to the real sequencing reads with RCA, and the simulated reads were approximate to the complexity of real sequencing data. Training data from previous studies [21] were used to calibrate the KNN model, ensuring consistency between simulated and empirical eccDNA length distributions. PBSIM3 [33] software was employed to generate long‐read sequencing data with parameters: ‐‐strategy wgs ‐‐method qshmm ‐‐qshmm QSHMM‐ONT‐HQ. model. To assess performance across varying sequencing depths, datasets were simulated at gradients coverage of 5×, 10×, 15×, 20×, 25×, 30×, 35×, 40×, 45×, and 50×. Additionally, we also employed the online available simulated datasets [17, 22] to comprehensively evaluate the eccDNA analysis pipelines.

### Statistical analysis

All statistical analyses were conducted using R packages. Wilcoxon rank‐sum test (equivalent to Mann–Whitney *U* test) is used for between‐group comparisons, Wilcoxon signed‐rank test is used for paired data and one‐sided tests, Holm–Bonferroni adjustment is used for multiple hypothesis testing in both between‐group and paired comparisons, and Pearson correlation is used for relationships assessing between variables. All analyses were reported in the corresponding figure legends with the test type, null hypotheses, and adjustment methods to ensure reproducibility. Differences were considered significant at *p* < 0.05.

## AUTHOR CONTRIBUTIONS

**Wang Li, Biyuan Miao, Jun Zhang, Qingsong Zeng, Zetong Wu**, and **Lilu Guo:** Software; data curation; formal analysis; visualization. **Biyuan Miao, Tangxuan Zhang, Yusheng Song, Mengting Li, Juan Luo**, and **Jingxiang Xu:** Methodology; investigation; resources. **Shaogui Wan, Wang Li**, and **Biyuan Miao:** Writing—original draft. **Shaogui Wan, Tianliang Liu, Shifu Chen**, and **Jia Gu:** Writing—review & editing. **Shaogui Wan:** Supervision. All authors have read the final manuscript and approved it for publication.

## CONFLICT OF INTEREST STATEMENT

The authors declare no conflicts of interest.

## ETHICS STATEMENT

No animals or humans were involved in this study.

## AI STATEMENT

ChatGPT‐5.1 was used for grammatical proofreading of the whole manuscript. All revisions were reviewed and validated by the authors and did not affect the data analysis methods, results, or conclusions.

## Supporting information


**Figure S1.** Sanger sequencing signal graphs.
**Figure S2.** Multi‐dimensional evaluation of the ECCFP performance using two online available simulated datasets.
**Figure S3.** Evaluation of the ECCFP performance using three simulated datasets with relaxed criteria.
**Figure S4.** The performance evaluation of ECCFP using simulated datasets with the coordinate homology.

**Table S1.** Chromosome position identified by eccDNA analysis pipelines and validated by Sanger sequencing.
**Table S2.** Results of the multi‐dimensional evaluation of eccDNA analysis pipelines with simulated datasets.
**Table S3.** Summary of public datasets for ECCFP evaluation.
**Table S4.** Summary of eccDNA identification from public data.
**Table S5.** Comparison of four eccDNA analysis pipelines.
**Table S6.** Primers for eccDNA validation.

## Data Availability

ECCFP is accessible at https://github.com/WSG-Lab/ECCFP. All analysis scripts and simulated datasets in this study are available at https://github.com/WSG-Lab/ECCFP_Project_Script. The simulated datasets generated in this study can be accessed at https://zenodo.org/records/17708118. Supplementary materials (figures, tables, graphical abstract, slides, videos, Chinese translated version, and update materials) may be found in the online DOI or iMetaOmics http://www.imeta.science/imetaomics/. Sequencing data have been deposited in the Genome Sequence Archive [34] in the National Genomics Data Center [35], China National Center for Bioinformatics/Beijing Institute of Genomics, Chinese Academy of Sciences under accession number PRJCA052047, which are available at https://ngdc.cncb.ac.cn/bioproject/browse/PRJCA052047.
