# Supplementary material for: ECCFP: A consecutive full pass‐based bioinformatic analysis for eccDNA identification from long‐read sequencing data
Source: IMetaOmics. 2026 Feb 3;3(1):e70080. doi: 10.1002/imo2.70080 (PMC13674448; doi:10.1002/imo2.70080)
Supplement: Supplementary file 1 [file IMO2-3-e70080-s001.docx]

Supporting information to:

**ECCFP: a consecutive full pass based bioinformatic analysis for eccDNA identification from long-read sequencing data**

**Running title**: ECCFP identifies eccDNA from long-read sequencing data

Wang Li^1,2#^, Biyuan Miao^1,3#^, Jun Zhang^1#^, Qingsong Zeng^1#^, Tangxuan Zhang^1#^, Zetong Wu^1^, Yusheng Song^4^, Mengting Li^5^, Lilu Guo^6^, Juan Luo^3^, Jingxiang Xu^3^, Tianliang Liu^7^, Shifu Chen^7^, Jia Gu^8^, Shaogui Wan^1*^

^1^Institute of Genomics and Precision Medicine, School of Medical Technology, Gannan Medical University, Ganzhou 341000, China

^2^School of Medical and Information Engineering, Gannan Medical University, Ganzhou 341000, China

3School of Basic Medicine, Gannan Medical University, Ganzhou 341000, China

^4^Department of Interventional Radiology, Ganzhou Hospital-Nanfang Hospital, Southern Medical University (Ganzhou People's Hospital), Ganzhou 341000, China

^5^Institute of Neurology, Jiangxi Academy of Clinical Medical Sciences, The First Affiliated Hospital, Jiangxi Medical College, Nanchang University, Nanchang 330006, China

^6^Department of Computer Science, Xidian University, Xi’an 710071, China

^7^LifeX Institute, School of Medical Technology, Gannan Medical University, Ganzhou 341000, China

^8^Department of Data Science, City University of Macau, Macau 999078, China

^#^These authors contributed equally: Wang Li, Biyuan Miao, Jun Zhang, Qingsong Zeng, Tangxuan Zhang

*Correspondence: [wansg@gmu.edu.cn](mailto:wansg@gmu.edu.cn) (Shaogui Wan)

**Figure S1.** Sanger sequencing signal graphs. (A) Sanger sequencing signal graph of eccDNA junction sequence identified in the HepG2 cell line. (B) Sanger sequencing signal graph of eccDNAs junction sequence identified in the spik-in eccMIRs sample.

**Figure S2.** Multi-dimensional evaluation of the ECCFP performance using two online available simulated datasets. (A) Assessment of the proportions of five reads types in two publicly available simulated datasets. We set stricter standards of 99% sequence homology and allowed a terminal deviation of 10 bp for a more comprehensive evaluation of data from Wanchai et al. 2022 (B) and Gao et al. 2024 (C).

**Figure S3.** Evaluation of the ECCFP performance using 3 simulated datasets with relaxed criteria. Under relaxed conditions of 90% CCS homology and a 20 bp terminal deviation, the evaluation includes: Multi-dimensional performance assessment (A) and false positive rate evaluation (B) among pipelines using simulated data constructed in this study. Multi-dimensional performance of the four pipelines was assessed (C), and their false positive rates were evaluated (D) using simulated data constructed from Wanchai et al. 2022. (E) The multi-dimensional performance of the four pipelines was assessed using simulated data constructed from Gao et al. 2024.

**Figure S4.** The performance evaluation of ECCFP using simulated datasets with the coordinate homology. Multi-dimensional performance assessment (A) and false positive rate evaluation (B) among four pipelines under relaxed conditions. Multi-dimensional performance assessment (C) and false positive rate evaluation (D) among four pipelines under strict conditions.
